# Integrating GWAS and eQTL analysis to decipher genetic mechanisms of feed efficiency and feeding behaviors in pigs

**DOI:** 10.1186/s12711-026-01048-7

**Published:** 2026-04-26

**Authors:** Zhenyang Zhang, Yongqi He, Wei Zhao, Ran Wei, He Han, Quanjun Zhan, Pengfei Yu, Sisi Li, Xiaoliang Hou, Jianlan Wang, Qingbo Zhao, Yan Fu, Zitao Chen, Zhen Wang, Yuchun Pan, Qishan Wang, Zhe Zhang

**Affiliations:** 1 Zhejiang Key Laboratory of Nutrition and Breeding for High-Quality Animal Products, College of Animal Sciences, Zhejiang University, Hangzhou, 310058 People’s Republic of China; 2 Xianghu Laboratory, Hangzhou, 311231 People’s Republic of China; 3 SciGene Biotechnology Co., Ltd, 1111# Luzhou Avenue, Hefei, 230000 People’s Republic of China; 4 School of Agriculture and Biology, Shanghai Jiao Tong University, 800# Dongchuan Road, Shanghai, 200240 People’s Republic of China; 5 College of Animal Science and Technology, Nanjing Agricultural University, Nanjing, 210095 People’s Republic of China

## Abstract

**Background:**

Feed constitutes the largest cost in pig farming. However, the genetic mechanisms underlying feeding behavior (FB) and feed efficiency (FE), as well as their relationships, remain poorly understood. This study aims to: (1) identify genetic variants associated with FB, FE, and production traits, while improving genomic prediction (GP) accuracy; (2) investigate the relationships between FB, FE, and production traits; and (3) explore potential links between pig feeding-related traits and human health traits.

**Results:**

A total of 358 lead SNPs associated with 28 feeding-related traits were identified through genome-wide association studies (GWAS) in 6938 genotyped pigs. In addition, GP was applied to an independent later-born population to validate the reliability of the GWAS summary statistics. By selection SNPs based on the P-values and linkage disequilibrium (LD), GP accuracy was improved by an average of 13.4%. Furthermore, a pleiotropic QTL located at SSC1:159,658,890–160,953,032 was identified for FB, FE, and production traits. Utilizing the cis-eQTL data from 34 tissues, seven trait-tissue-gene associations were identified. Additionally, mendelian randomization analysis indicated that increased feed intake per visit was genetically correlated with both FE and average daily gain, and eating more in the night might reduce lean meat percentage. Notably, heritability enrichment analysis revealed that GWAS signals for pig feeding behavior traits were significantly enriched in genomic regions associated with human fat intake–related traits.

**Conclusion:**

This study identified genetic variants associated with FE, FB, and production traits, and clarified their causal relationships. It represents the first elucidation of the connection between pig feeding traits and human traits. These insights are crucial for molecular breeding in pigs and for evaluating the suitability of pigs as model organisms in biomedical research.

**Supplementary Information:**

The online version contains supplementary material available at 10.1186/s12711-026-01048-7.

## Background

Pigs are not only a major source of meat, accounting for 40% of meat intake globally, but also serve as crucial animal models in medical research, such as studies on human diseases and xenotransplantation [1]. Feed accounts for 65% of total pork production costs [2, 3], exacerbating competition for food resources between humans and livestock and threatening food security. Despite this, research on the genetic mechanisms underlying pig feed-related traits remains limited, hindering genetic improvement of feed efficiency (FE) and leaving the connection between pig feeding behaviors (FB) and human dietary behaviors largely unexplored.

Elucidating the genetic mechanisms underlying FE traits can facilitate molecular breeding strategies, such as marker-assisted selection and genomic selection (GS), to achieve genetic improvement in FE. Previous studies have identified candidate genes and variants associated with FE traits in pigs through genome-wide association studies (GWAS). For instance, using data on 1365 boars, Li et al. [4] identified 35 candidate genes, including *MC4R* and *INSR*, to be associated with FE related traits. Guo et al. [5], using data on 1912 Duroc × Erhualian F2 pigs, identified six chromosomal regions associated with FE related traits. However, these studies are limited by small sample sizes, restricting the potential discoveries and applications of GWAS. Additionally, many the identified associated variants reside in noncoding regions and may be in high linkage disequilibrium (LD) with causal variants, complicating the interpretation of their molecular mechanisms. To address this, increasing GWAS population diversity has been suggested as a method to reduce the impact of LD on fine mapping [6]. In humans, numerous traits or disease-associated signals have been identified by cross-ancestry GWAS [7, 8] [needs reference]. For instance, multi-ancestry GWAS identified 53 novel loci for major depression [8] and 35 new susceptibility loci for atrial fibrillation [7]. In addition, integrating molecular QTL (molQTL) with GWAS findings is also effective for elucidating the roles of these signals, and has been used to decipher the genetic variants of complex traits or disease in humans [7, 9], pigs [10, 11], and cattle [12].

Previous studies have revealed associations between FB and FE at both phenotypic and genetic levels. At the phenotypic level, FB such as fewer meals per day [13] and faster eating rates [2] were reported to enhance FE in pigs. However, conflicting results have also been observed. For example, based on the genetic correlation analysis, fast-eating pigs were found less efficient [14, 15]. These findings highlight the complexity of their relationship and the need for more data to achieve robust conclusions. Recently, Mendelian randomization (MR) has effectively been used to clarify the genetic relationships between phenotypes using individual or summary level data [16]. For instance, Timasheva et al.[17] demonstrated that genetically predicated eating patterns influence both anthropometric and metabolic traits in humans. Despite these advances, the application of MR to pig FB traits remains limited due to the lack of GWAS summary data from large samples.

Furthermore, pigs and humans share remarkable similarities in digestive tracts [18, 19] and nutritional requirements as omnivores [20]. However, humans exhibit other eating patterns compared to pigs, such as emotional and restrained eating, which are associated with an increased risk of obesity, metabolic syndrome and related complications [21]. One of the challenges in studying human dietary behavior is data collection, as it relies on subjective questionnaires and it is almost impossible to observe patients continuously for 24 h or conduct physical interventions. Pigs have been used in research on human diet-related diseases. For example, studies have used pigs to investigate food conditioning and the brain reward circuit’s response to flavors with positive or negative hedonic values [22, 23]. Despite their potential, the extensive data that is systematically recorded in pig breeding practices through automated feeding devices, such as body weight, feed intake, and FB remain underappreciated. These data could be helpful when studying human eating behaviour, obesity, circadian rhythms, and other behavior phenotypes.

In this study, we collected FB data from 26,115 pigs and genetically sequenced 6,938 individuals across a range of commercial breeds, including Duroc, Yorkshire, Landrace, Pietrain, and Duroc2 (another Duroc line characterized by high intramuscular fat content [24]). We conducted GWAS for 28 feeding-related traits and further analyzed the data using molQTL from FarmGTEx and GWAS summary statistics from 205 human phenotypes in the GWAS Catalog. Our study aimed to: (1) identify markers associated with FB, FE, and production traits in pigs, and utilize the findings to improve GP accuracy; (2) investigate the causal relationship between FB, FE, and production traits; and (3) identify human traits that are associated with the pig feeding-related traits. These results can drive advancements in breeding for FE in pigs and are expected to catalyze more studies linking pig phenotypes to human traits.

## Material and methods

### Experimental population and dataset

Data for this study were collected from pigs raised at a nucleus farm of SciGene Biotechnology company (Guangxi, China) between 2019 and 2023. A total of 26,115 pigs from various breeds were included: Duroc (DD; n = 4589), Landrace (LL; n = 4705), Yorkshire (YY; n = 12,925), Pietrain (PP; n = 2529), and Duroc2 (D2; n = 1367).

Pigs were randomly selected to participate in a feeding test, starting at approximately 17 weeks of age (115.7 ± 4.2 days) and ending at around 24 weeks of age (164.7 ± 4.2 days). During this period, they were housed in groups averaging 15 animals per pen and were monitored using a single-space automatic feeding system (9ZC-170 Intelligent Swine Growth Performance Monitoring System, Guangdong Guangxing Livestock Machinery Equipment Co., Ltd.) across 169 pens distributed in 4 herds. Pigs were provided ad libitum access to feed and subjected to the same management.

Additionally, pig weights were measured manually at the beginning (OnsetWeight, 67.5 ± 8.9 kg) and end (FinalWeight, 115.8 ± 11.4 kg) of the test period, independent of the automatic feeder system. At the end of the test, backfat thickness (BFT) and loin muscle depth (LMD) were assessed via ultrasound between the 3rd and 4th last rib using EXAGO. Meanwhile, a total of 7934 pigs were randomly selected to scan body using a CT device (Siemens AS plus) with settings of 110 kV/160 mA, matrix 512 × 512, axial, 4.99 mm thickness. The live lean meat percentage (LP) of the carcass was quantified as described by Pan et al. [25], who developed a bidirectional convolutional residual framework to segment and remove the internal organs, then divide and quantify the composition of live pigs in CT scans automatically. Lean body mass was calculated as: Lean = LP × (FinalWeight—OnsetWeight).

### Data editing and traits definition

Each visit to the feeder was recorded, capturing start and end times, feed intake, pig weight, and pig ID, resulting in 23,009,702 feeder visits logged across all pigs. Records with test ages below 100 or above 200 days were excluded from the dataset. Records with body weights exceeding 180 kg or less than 25 kg were also removed. Further data cleaning was performed following the guidelines of Casey et al. [26]. After data editing, a total of 22,800,656 records related to feed intake and feeding time were retained for further analysis. This study focused on three groups of traits: FE, FB, and off-test production traits. A total of 28 traits were included, comprising 25 classical traits, hourly feed intake traits, hourly feeding time traits, and hourly feeding frequency traits, as detailed in Table 1. All trait definitions and calculation procedures are provided in Table 1; in general, traits were summarized over the entire test period unless otherwise specified.

**Table 1:** Trait definitions and models

Category	Trait	Definition	Model or measurements
Production trait	ADG (kg)	Average daily gain	The slope of body weight regressed on age in days
	BFT (mm)	Backfat thickness	Ultrasound
	LMD (mm)	Lean muscle depth	Ultrasound
	IMF (%)	Intramuscular fat content	Ultrasound
	LP (%)	Lean meat percentage by CT scan	Computed Tomography
FB	DFI (kg)	Feed intake per day	Total of feed intake / number of days in the experiment
	NVD (times)	Number of visits to feeder per day	Total of visits / number of days in the experiment
	TPD (min)	Total time spent eating per day	Total of eating time/ number of days in the experiment
	DFI_night (%)	Proportion of feed intake during the period from 8:00 PM to 4:00 AM, relative to the total daily feed intake	Total of feed intake in 8 PM to 4 AM/ Total of number of visits
	NVD_night (%)	Proportion of feeding events occurring between 8:00 PM and 4:00 AM, relative to the total daily feeding events	Total of number of visits in 8 PM to 4 AM/Total number of visits
	TPD_night (%)	Proportion of feeding time occurring between 20:00 and 4:00, relative to the total daily feeding time	Total of eating time in 8 PM to 4 AM/ Total of number of visits
	FPV (kg)	Feed intake per visit	DFI/NVD
	TPV (min)	Total time spent eating per visit	TPD/NVD
	FR (kg/m)	Feed rate	FPV/TPV
	FIH (g)	Feed intake per hour (measured from 00:00 to 23:00)	
	TPH (min)	Feeding time per hour (measured from 00:00 to 23:00)	
	NVH (times)	Number of visit per hour (measured from 00:00 to 23:00)	
	FI235(%)	Proportion of visits where the feed intake exceeded 235 g relative to the total number of visits to feeder	Number of visits which feed intake more than 235 g / Total of number of visits
	FI23(%)	Proportion of visits where the feed intake below 23 g relative to the total number of visits to feeder	Number of visits which feed intake less than 23 g / Total of number of visits
FE	FCR (kg/kg)	Feed Conversion Ratio	DFI/ ADG
	RFI_ADG (kg)	Residual feed intake	DFI ~ Sex + Breed + Pen + ADG + e
	RFI_ADG_MBW (kg)	Residual feed intake	DFI ~ Sex + Breed + Pen + ADG + MBW + e
	RFI_ADG_BF (kg)	Residual feed intake	DFI ~ Sex + Breed + Pen + ADG + BF + e
	RFI_ADG_BF_MBW (kg)	Residual feed intake	DFI ~ Sex + Breed + Pen + ADG + MBW + BF + e
	RFI_ADG_BF_MD (kg)	Residual feed intake	DFI ~ Sex + Breed + Pen + ADG + BF + LMD + e
	RFI_ADG_BF_MBW_MD (kg)	Residual feed intake	DFI ~ Sex + Breed + Pen + ADG + MBW + BF + LMD + e
	RFI_CT_lean (kg)	Residual feed intake	DFI ~ Sex + Breed + Pen + ADG + MBW + Lean + e
	RDG (kg)	Residual body weight daily gain	ADG ~ Sex + Breed + Pen + DFI + MBW + e

We identified two peaks in the distribution of pig feed intake per visit, at 23 g and 235 g (Additional file 2, Figure S1). Based on this, we calculated the proportion of meals with feed intake below 23 g (FI23) and above 235 g (FI235) across all recorded feeding events, classifying these as “small meal” and “large meal” pigs, respectively.

Feed efficiency traits, including feed conversion ratio (FCR), residual feed intake (RFI), and residual daily gain (RDG), were calculated as described in Table 1. Briefly, FCR was defined as the ratio of daily feed intake (DFI) to average daily gain (ADG). RFI was estimated as the residual from linear regression models of DFI on production and body composition traits, accounting for fixed effects such as sex, breed, and pen. RDG was similarly defined as the residual from a regression model of ADG on DFI and other covariates. The detailed model specifications and all covariates included in each model are provided in Table 1. In all models, the residual term represents the difference between observed and predicted values. The descriptive statistics of the phenotypes are presented in Additional file 1, Table S1.

### Estimation of genetic parameters

A univariate animal model was applied to estimate the heritability of each trait within each breed. The model was specified as follows:

1 $$ {\mathrm{y}} = {\mathrm{Xb}} + {\mathrm{Za}} + {\mathrm{Vp}} + {\mathrm{e}} $$

where $\mathbf{y}$ is the vector of observations (FE, FB and production traits); $\mathbf{b}$ is the vector of fixed effects including overall mean, sex (2 levels), parity (10 levels); $\mathbf{a}$ is the vector of additive genetic effects of the animal, assumed to follow normal distribution N(0,${\mathbf{A}}\sigma_{a}^{2}$), where **A** is the numerator relationship matrix constructed from pedigree spanning three generations; $\mathbf{p}$ is the random group effects (combining by onset-herd, onset year and season, 2,063 levels), with $\mathbf{p}$ ∼ N (0, **I**${\sigma }_{p}^{2}$), season was defined according to the Northern Hemisphere calendar, with March–May as the first quarter, June–August as the second quarter, September–November as the third quarter, and December–February as the fourth quarter; $\mathbf{e}$ is the vector of random residuals, with *N*(0, **I**${\sigma }_{e}^{2}$); **X, Z** and **V** are the corresponding incidence matrices. Due to the consideration of sex, breed, and group effects in the calculation process for traits RFIs, and RDG, heritability was estimated for these traits by only considering the fixed effects of parity and a mean.

Variance components were estimated by average information restricted maximum likelihood (AI-REML) method implemented in DMU software package (https://dmu.ghpc.au.dk/dmu/index.html) [27]. The heritability (h^2^) was computed as:

2 $$ h^{2} = \frac{{\sigma_{a}^{2} }}{{\sigma_{a}^{2} + \sigma_{p}^{2} + \sigma_{e}^{2} }} $$

The vector of corrected phenotypes for each trait was calculated as:

3 $$ {\boldsymbol{y}}^{\user2{*}} = \hat{\user2{\mu }} + \hat{\user2{a}} + \hat{\user2{e}} $$

where $\widehat{{\boldsymbol{\mu}}}$ is the estimate of the overall mean and $\widehat{{\boldsymbol{a}}}$ and $\widehat{{\boldsymbol{e}}}$ are estimated breeding values and residuals, respectively.

Genetic correlations were estimated using the following bivariate model for each pair of traits to reduce computational burden:

4 $$ \left[ {\frac{{{\boldsymbol{y}}_{{\boldsymbol{m}}} }}{{{\boldsymbol{y}}_{{\boldsymbol{n}}} }}} \right] = \left[ {\frac{{{\boldsymbol{X}}_{{\boldsymbol{m}}} }}{0}\frac{0}{{{\boldsymbol{X}}_{{\boldsymbol{n}}} }}} \right]\left[ {\frac{{{\boldsymbol{b}}_{{\boldsymbol{m}}} }}{{{\boldsymbol{b}}_{{\boldsymbol{n}}} }}} \right] + \left[ {\frac{{{\boldsymbol{Z}}_{{\boldsymbol{m}}} }}{0}\frac{0}{{{\boldsymbol{Z}}_{{\boldsymbol{n}}} }}} \right]\left[ {\frac{{{\boldsymbol{a}}_{{\boldsymbol{m}}} }}{{{\boldsymbol{a}}_{{\boldsymbol{n}}} }}} \right] + \left[ {\frac{{{\boldsymbol{V}}_{{\boldsymbol{m}}} }}{0}\frac{0}{{{\boldsymbol{V}}_{{\boldsymbol{n}}} }}} \right]\left[ {\frac{{{\boldsymbol{p}}_{{\boldsymbol{m}}} }}{{{\boldsymbol{p}}_{{\boldsymbol{n}}} }}} \right] + \left[ {\frac{{{\boldsymbol{e}}_{{\boldsymbol{m}}} }}{{{\boldsymbol{e}}_{{\boldsymbol{n}}} }}} \right] $$

where ${\boldsymbol{y}}$, ${\boldsymbol{X}}$, ${\boldsymbol{b}}$, ${\boldsymbol{Z}}$, ${\boldsymbol{a}}$, ${\boldsymbol{V}}$, ${\boldsymbol{p}}$**,** and ${\boldsymbol{e}}$ are identical to Eq. (1) and the subscripts $m$ and $n$ represent the $m\mathrm{th}$ and $n\mathrm{th}$ traits. In this model, ${\boldsymbol{a}}$**=**$\left[\frac{{{\boldsymbol{a}}}_{{\boldsymbol{m}}}}{{{\boldsymbol{a}}}_{{\boldsymbol{n}}}}\right]$ is assumed to follow a bivariate normal distribution N(0,$\left[ {\frac{{\sigma_{a\left( m \right)}^{2} }}{{\sigma_{{a\left( {m,n} \right)}} }}\frac{{\sigma_{{a\left( {m,n} \right)}} }}{{\sigma_{a\left( n \right)}^{2} }}} \right] \otimes {\boldsymbol{A}}$) and ${\boldsymbol{e}}= \left[\frac{{{\boldsymbol{e}}}_{{\boldsymbol{m}}}}{{{\boldsymbol{e}}}_{{\boldsymbol{n}}}}\right]$ is assumed to follow a bivariate normal distribution N(0,$\left[ {\frac{{\sigma_{e\left( m \right)}^{2} }}{0}\frac{0}{{\sigma_{e\left( n \right)}^{2} }}} \right] \otimes {\boldsymbol{I}}$, where ${\sigma }_{a(m,n)}$ is the genetic covariance between $m$ and $n$, ${\sigma }_{a(m)}^{2}$ and ${\sigma }_{a(n)}^{2}$ are the genetic variances of $m$ and $n$, respectively, ${\sigma }_{e(m)}^{2}$ and ${\sigma }_{e(n)}^{2}$ are the residual variances of $m$ and $n$, respectively, and $\otimes$ is the Kronecker product.

The genetic correlations were calculated as:

5 $$ r_{{g\left( {m,n} \right)}} = \frac{{\sigma_{{a\left( {m,n} \right)}} }}{{\sqrt {\sigma_{a\left( m \right)}^{2} \sigma_{a\left( n \right)}^{2} } }} $$

### Genotypic data

A total of 6938 pigs were with low genetic relatedness were selected and genotyped using low-coverage sequencing (1 ~ 2 $\times$). The pigs were divided into discover and validation groups by birth date. The validation group consisted of 1200 younger pigs, including 500 YY, 400 DD, and 300 LL pigs, while the remaining 5738 pigs formed the discovery group. A total of 23 pigs were randomly selected and genotyped based on a SNP array (Neogen, GGP 50 K Porcine v1 Genotyping BeadChip) to assess imputation accuracy.

Ear tissues were collected from the pigs for DNA extraction and library generation. The libraries were sequenced using the BGI platform (BGISEQ, DNBSEQ-T7, PE 150 model, 3G/pigs). Raw short sequencing reads were filtered using fastp (version 0.23.1) [28]. Sequencing reads from the low-coverage samples were mapped to the Sscrofa11.1 reference genome [29] using GTX [30], producing BAM files and removing PCR duplicates. Coverage and depth for each individual were calculated using Mosdepth (version 0.3.2) [31].

### Evaluation of genotype imputation

GLIMPSE v2 [32] was used to impute SNPs with the PHARPv3 panel [33]. Genotypes with INFO scores below 0.3 were excluded (Additional file 2, Figure S2A). INFO scores showed a positive correlation with increasing minor allele frequency (MAF), and after excluding SNPs with MAF below 0.05, all remaining SNPs had INFO scores exceeding 0.9 (Additional file 2, Figure S2B). Sex chromosomes were excluded (Additional file 2, Figure S2E). The concordance rate (CR) compared to genotypes from the SNP array reached 96.5% (Additional file 2, Figure S2C) and improved with increasing sequencing depth (Additional file 2, Figure S2D). Finally, genotypes at 12,497,601 SNPs in 18 autosomes on 6938 pigs across five breeds [DD (n = 1,465), LL (n = 1,376), YY (n = 2,511), PP (n = 791), and D2 (n = 795)] (Additional file 2, Figure S2F) were retained for analysis.

### Genome-wide association study

The GWAS was conducted using the fastGWA [34] method implemented in GCTA [35] (v1.94.0) using the discover population (n = 5738), using the following statistical mode:

6 $$ y^{*} = 1\mu + Pf + X\beta + Zg + e $$

where ${\mathbf{y}}^{\mathbf{*}}$ is a vector of corrected phenotypes from model (3); **μ** is the overall mean; **f** is the top 10 principal components extracted from the genotypes using GCTA [35]; $\upbeta$ is the allele substitution effect for the SNP analyzed (the model was run separately for each SNP); $\mathbf{X}$ is a vector of marker genotypes, coded as 0, 1 and 2 for A_1_A_1_, A_1_A_2_ and A_2_A_2_ genotypes, respectively; $\mathbf{P}$ and $\mathbf{Z}$ are the design matrices; $\mathbf{g}$ is the random polygenic effect assumed to follow the distribution $\mathbf{g}$ ~ N(0, **G** ${\upsigma }_{g}^{2}$), where **G** is the genomic relationship matrix and ${\upsigma }_{g}^{2}$ is the additive genetic variance; ${\boldsymbol{e}}$ is the random residual effect with ${\boldsymbol{e}}$ ~ N(0, **I** ${\upsigma }_{\mathrm{e}}^{2}$), where **I** is an identity matrix and ${\upsigma }_{\mathrm{e}}^{2}$ is the residual variance. Parameters ${\upsigma }_{g}^{2}$ and ${\upsigma }_{\mathrm{e}}^{2}$ were estimated by the grid-search-based REML algorithm. The **G** matrix was constructed as follows [36]:

7 $${\mathrm{G}} = {\mathrm{MDM}}^{\prime} $$

where $\mathbf{M}$ is the genotype matrix for the SNPs, elements of which are coded as 0–2 ${p}_{i}$, 1–2 ${p}_{i}$, 2–2 ${p}_{i}$ for A_1_A_1_, A_1_A_2_ and A_2_A_2_ genotypes, respectively, and ${p}_{i}$ is the frequency of allele A_2_ for the *i*th locus, and $\mathbf{D}$ is a diagonal matrix with ${D}_{ii}= \frac{1}{m[2{p}_{i}(1-{p}_{i})]}$, where $m$ is the number of SNPs.

The *p*-value for each SNP’s allele substitution effect was calculated using a Wald test. To account for multiple testing, we applied a Bonferroni correction by setting the genome-wide significance threshold to 0.05/n, where n is the total number of SNPs included in the GWAS, and the suggestive significance threshold to 1/n,.

### Impact of GWAS findings on genomic prediction accuracy

To evaluate the impact of GWAS findings on genomic prediction (GP) accuracy, we conducted 10 repetitions of tenfold cross-validation in the validation population (n = 1200). In each repetition, the population was randomly divided into 10 groups, with nine groups designated as the reference population and one group as the test dataset (masked phenotypes). After each iteration, GP accuracy was assessed by Pearson’s correlation between the genomic estimated breeding values (GEBV) obtained from tenfold cross-validation using GBLUP and estimated breeding values (EBV) derived from pedigree-based BLUP (PBLUP) fitted using the full dataset, including all phenotypic records.

The GBLUP model used in the validation population was:

8 $$ {\mathbf{y}}^{*} = {\mathbf{u}} + {\mathbf{Za}} + {\mathbf{e}} $$

Where y* is the corrected phenotype from model (3); **u** is the mean; ***a*** is the additive genetic effect, assumed to follow a normal distribution N(0, **G** ${\upsigma }_{\mathrm{a}}^{2}$), where **G** is the numerator relationship matrix constructed from selected SNPs and ${\sigma }_{a}^{2}$ is the additive genetic variance; ***e*** is the vector of random residuals, with N(0, **I** ${\upsigma }_{\mathrm{e}}^{2}$), where **I** is an identity matrix; **Z** is the corresponding incidence matrix.

Selection of loci for GP from GWAS summary statistics remains an unresolved issue. To evaluate the impact of GWAS-based marker selection on the accuracy of GP, we constructed multiple SNP subsets using different filtering strategies. All SNP selection strategies are comprehensively summarized in Table 2. Briefly, Independent SNPs were identified using PLINK with an LD threshold of r^2^ = 0.4 and *P* values < 0.01 (“Clump” subset) separately for each trait. Additional subsets were generated by selecting SNPs with association GWAS *P* values below 0.01 (“*P*value001”) or 0.001 (“*P*value0001”). To obtain uniformly distributed loci across the genome, the genome was divided into segments of 25 Kb, 50 Kb, 100 Kb, 300 Kb, or 1000 Kb, and the SNP with the smallest *P* values in each segment was retained (“Uniform” subsets). Furthermore, for cross-trait evaluation, we combined loci meeting the above criteria from all traits or specific trait categories, including feed behavior (FB), feed efficiency (FE), and production traits, to generate combined subsets (e.g., “Combine_001”, “Combine_behavior_clump”).

**Table 2:** Summary of SNP subsets used for genomic prediction (GP)

SNP subset	Definition
Clump	Independent SNPs selected based on LD threshold (r^2^ < 0.4) and significance level (*P* < 0.01) using PLINK
Pvalue001	SNPs with GWAS* P* < 0.01
Pvalue0001	SNPs with GWAS *P* < 0.001
Uniform25k	Genome divided into 25 Kb windows; the most significant SNP (GWAS *P* value) within each window was selected
Uniform50k	Genome divided into 50 Kb windows; the most significant SNP in each window was selected
Uniform100k	Genome divided into 100 Kb windows; the most significant SNP in each window was selected
Uniform300k	Genome divided into 300 Kb windows; the most significant SNP in each window was selected
Uniform1000k	Genome divided into 1000 Kb windows; the most significant SNP in each window was selected
Combine_001	Combined SNPs with *P* < 0.01 from all traits
Combine_0001	Combined SNPs with *P* < 0.001 from all traits
Combine_clump	Combined independent SNPs (LD r^2^ < 0.4, *P* < 0.01) from all traits
Combine_behavior_001	Combined SNPs with *P* < 0.01 from all feed behavior traits
Ccombine_behavior_0001	Combined SNPs with *P* < 0.001 from all feed behavior traits
Combine_behavior_clump	Combined independent SNPs from all feed behavior traits
Combine_rfi_001	Combined SNPs with *P* < 0.01 from all feed efficiency traits
Combine_rfi_0001	Combined SNPs with *P* < 0.001 from all feed efficiency traits
Combine_rfi_clump	Combined independent SNPs from all feed efficiency traits
Combine_production_001	Combined SNPs with *P* < 0.01 from all production traits
Combine_production_0001	Combined SNPs with *P* < 0.001 from all production traits
Combine_production_clump	Combined independent SNPs from all production traits

Each subset was constructed from GWAS summary statistics using different selection criteria based on *P* value thresholds, LD pruning, or genome-wide uniform segmentation

The GBLUP and PBLUP were run using the DMU software package (https://dmu.ghpc.au.dk/dmu/index.html) [27].

### Post GWAS analysis

#### Identification of independently associated genetic variants

To identify lead SNPs, which are significantly associated with the trait after conditioning on other associated variants within the same genomic region. This was achieved by the stepwise model selection algorithm (cojo-slct) from GCTA with default parameters [37]. Briefly, this approach iteratively selects the most significant SNP and then re-tests the remaining SNPs conditional on those already included in the model, thereby identifying multiple independently associated loci within a region. The linkage disequilibrium (LD) reference was generated using the GWAS population.

#### Transcriptome-wide association study (TWAS)

To explore the association between gene expression levels and complex traits, we conducted Transcriptome-Wide Association Studies (TWAS) for 34 pig tissues using S-PrediXcan [38] implemented in the TWAS-server [39], which was designed for the FarmGTEx project [10, 12], allowing users to conveniently perform TWAS. Briefly, TWAS estimates the genetically regulated component of gene expression by integrating individual genotypes with pre-trained expression weights, which represent SNP–expression effect sizes derived from cis-eQTL models trained using Elastic Net in 34 tissues from the pigGTEx resource. And then tests the association between the predicted gene expression levels and phenotypic traits. The S-PrediXcan framework performs TWAS using GWAS summary statistics, yielding equivalent association results to individual-level TWAS analyses.

#### Colocalization between cis-eQTL and GWAS signals

To investigate the potential regulatory mechanism of genes affecting the traits, we conducted colocalization analysis using the coloc.abf function in the coloc [40] R package. This analysis was based on 1 Mb up- and downstream of the independent associated SNPs from the GWAS summary and the *cis*-eQTL data from PigGTEx [10]. The colocalization approach estimates the posterior probabilities of five hypotheses: PPH0 (no association with either trait), PPH1 (association with the first trait only), PPH2 (association with the second trait only), PPH3 (association with both traits but driven by different causal variants), and PPH4 (association with both traits driven by the same causal variant). We considered loci with PPH4 > 0.8 as having strong evidence for colocalization between gene expression and the trait, indicating a potential causal regulatory relationship.

#### Summary-data-based Mendelian randomization between phenotypes and eQTLs

To determine causality between gene expression and complex traits, we conducted MR analysis between *cis*-eQTL and GWAS. Because the GWAS and eQTL datasets were derived from two independent populations, we conducted two-sample MR using the SMR tool (version 1.03) [41]. The HEIDI (heterogeneity in dependent instruments) test was applied to distinguish pleiotropy from linkage using multiple SNPs within a *cis*-eQTL region. We defined significance with thresholds of *P* < 0.05/n and p_HEIDI > 0.05, where n is the number of genes tested in the tissue.

#### One-sample Mendelian randomization analysis

To investigate the causal effects from FB to FE and production traits, we performed one-sample MR analysis using individual data, as both phenotypes were measured in the same population and corresponding genotype data were available. This involves three steps. (1) Instrumental variants (IVs) selection: SNPs were selected as IVs with a threshold of *P* < 1 × 10^−5^ based on GWAS and LD r^2^ < 0.001 for each trait. To exclude weak IVs, the strength of each IV was evaluated based its association with the exposure trait, and SNPs with an F-statistics < 10 were removed. To address pleiotropy, SNPs associated with the outcome traits at *P* < 1 × 10^−5^ were removed. (2) Two-stage least square regression (TSLS): TSLS analysis was performed using the ivreg function in the AER [42] R package. In the first stage, we assessed the association between the corrected phenotype and IVs for each FB trait using linear regression, and obtained predicted fitted values based on the instruments. In the second stage, we performed linear regression with the corrected phenotype of the FE and production trait and the genetically predicted exposure level from the first stage. Both stages were adjusted for the top five principal components (PCs). (3) Sensitivity analysis: We conducted a leave-one-out analysis for each IV and removed those that could potentially reverse the direction of the effects until all tests showed consistent directions.

### Comparative analysis between human and pig

We performed partitioned heritability analysis using stratified LD score regression (LDSC-seg), as described by Finucane et al. [43], to assess whether genomic regions associated with pig feeding-related traits are enriched for the heritability of human traits. We compiled GWAS summary data for 205 human traits (sample size > 10,000) from the GWAS Catalog [44]. These traits were grouped into 16 categories that are relevant to feeding behavior, body composition, metabolism, and related behavioral or lifestyle factors in humans, that may share biological pathways with pig feeding and production traits. The categories, derived from the catalog’s MAPPED_TRAIT, included fat intake, diet, fat body mass, body fat percentage, body mass index, body height, body weight, social environment, worry, self-injurious behavior, sleep disorder, eating behavior, blood protein, metabolite, vitamin supplement, and alcohol consumption.

Specifically, genomic regions spanning ± 1 Mb around associated variants of pig feeding-related traits were mapped to the human genome (GRCh38/hg38) using UCSC LiftOver [45], and used to construct binary annotation tracks.

Partitioned heritability was estimated using the stratified LD score regression model, where the expected χ^2^ statistic for SNP j is modeled as:

9 $$ E\left[ {\chi_{j}^{2} } \right] = 1 + N\mathop \sum \limits_{c} \tau_{c} \iota_{j,c} $$

where N is the sample size, ${\tau }_{c}$ represents the per-SNP contribution of annotation* c* to heritability, and ${\iota }_{j,c}$ is the LD score of SNP j with respect to annotation *c*.

The heritability explained by annotation *c* was calculated as:

10 $$ h_{c}^{2} = \mathop \sum \limits_{j \in c} \mathop \sum \limits_{c} \tau_{c} \iota_{j,c} $$

where ${\iota }_{j,c}$ represents the LD score of SNP j with respect to annotation c, and the summation is taken over all SNPs within annotation c.

The total SNP-based heritability (${h}^{2}$) was computed by summing contributions across all SNPs:

11 $$ h^{2} = ~\mathop \sum \limits_{j} \mathop \sum \limits_{c} \tau _{c} \iota _{{j,c}} $$

Enrichment was defined as the proportion of SNP-based heritability explained by SNPs within the annotation divided by the proportion of SNPs in that annotation:

12 $$ Enrichment = (h_{c}^{2} /h^{2} )/(M_{c} /M) $$

where ${h}_{c}^{2}$ is the heritability explained by SNPs in annotation c, *h*^*2*^ is the total SNP-based heritability, ${M}_{c}$ is the number of SNPs in the annotation, and *M* is the total number of SNPs. Statistical significance was assessed using the regression coefficient (${\tau }_{c}$) and its standard error estimated by LDSC. Annotations with enrichment fold > 1 and *P* < 0.05 were considered significantly enriched. Finally, we performed Fisher’s test using the fisher.test function in R to identify human trait categories that were enriched for genomic regions associated with pig traits.

### Functional annotation

Gene annotation was conducted using VEP [46]. GO and KEGG functional enrichment analyses were performed with KOBAS [47]. Detailed investigations were carried out using the PigBiobank [48] and Pig RNA Atlas [49].

## Results

### Phenotypic statistics

**Fig. 1:**
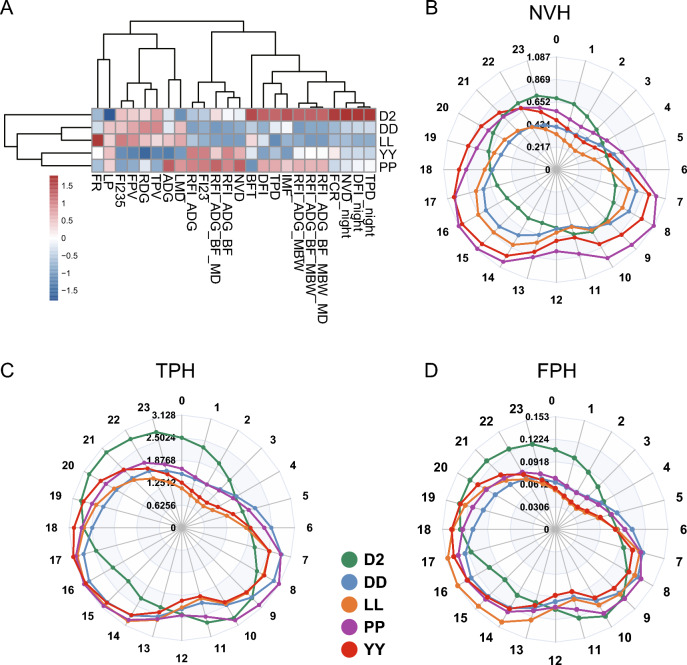
Comparison of phenotypes between FB traits, feed efficiency (FE), and production traits across pig breeds. **A** Heatmap showing the phenotypes of different traits across breeds. The x-axis represents traits, the y-axis represents breeds, and the color scale indicates standardized phenotypic values. **B** Radar chart illustrating the number of feeding visits at each hour. The outer circle represents the 24 hourly time points from 0 to 23, and the values on the radial axes indicate the average number of visits per breed at each hour. The green, blue, orange, purple, and red lines correspond to Duroc2, Duroc, Landrace, Pietrain, and Large White pigs, respectively. **C** Hourly feeding duration across the 24 time points. **D** Hourly feed intake across the 24 time points

A total of 28 traits were included in this study (Fig. 1a–d and Additional file 1, Table S1). We observed that the YY and PP breeds, as well as the DD and LL breeds, clustered together based on phenotypic traits, while D2 formed another separate branch (Fig. 1a). YY and PP exhibited higher NVD, FI23, and TPV compared to the other breeds, while DD and LL had higher FI235 and FPV, consistent with previous studies [49, 50]. Additionally, D2 showed higher RFIs, IMF, and night FB than the other breeds (Fig. 1a and Additional file 1, Table S1). Furthermore, D2 exhibited later peak feeding periods at around 9–11 AM and 8–10 PM, whereas the peak periods for DD, LL, PP, and YY were around 7–9 AM and 3–5 PM (Fig. 1b–d). On average, D2 had 30% of its feed intake, counts, and feeding time occurring at night, compared to approximately 20% for the other breeds (Additional file 1, Table S1).

### Estimated genetic parameters

The heritability estimates for FE, FB, and production traits ranged from 0.24 to 0.49, 0.15 to 0.59, and 0.21 to 0.71 (Table 3), respectively. For RFIs, heritability estimated varied depending on the adjustment methods. RFIs that considered MBW exhibited slightly lower heritability compared to those did not, as evidenced by paired t-tests (RFI_ADG vs RFI_ADG_MBW, *P* = 0.012; RFI_ADG_BF vs RFI_ADG_BF_MBW, *P* = 0.047). According to the Akaike information criterion (AIC), models that incorporated MBW provided a better fit to the data (Additional file 1, Table S2). Among all RFI definitions, RFI_CT_lean showed the lowest AIC value, indicating the best model performance (Additional file 1, Table S2).

**Table 3:** Estimates of heritabilities *h*^*2*^(SE) by breed

Trait	DD	LL	YY	PP	D2
ADG	0.33 (0.04)	0.33 (0.04)	0.39 (0.02)	0.31 (0.05)	0.38 (0.07)
RDG	0.43 (0.04)	0.39 (0.04)	0.42 (0.03)	0.34 (0.05)	0.36 (0.07)
FCR	0.24 (0.03)	0.36 (0.04)	0.29 (0.02)	0.23 (0.04)	0.27 (0.06)
RFI_ADG	0.43 (0.04)	0.46 (0.04)	0.43 (0.03)	0.49 (0.05)	0.38 (0.07)
RFI_ADG_MBW	0.38 (0.04)	0.4 (0.04)	0.4 (0.02)	0.48 (0.05)	0.32 (0.06)
RFI_ADG_BF	0.36 (0.04)	0.41 (0.04)	0.38 (0.02)	0.44 (0.05)	0.3 (0.06)
RFI_ADG_BF_MBW	0.34 (0.04)	0.37 (0.04)	0.35 (0.02)	0.44 (0.05)	0.29 (0.06)
RFI_ADG_BF_MD	0.35 (0.04)	0.41 (0.04)	0.38 (0.02)	0.43 (0.05)	0.29 (0.06)
RFI_ADG_BF_MBW_MD	0.34 (0.04)	0.37 (0.04)	0.35 (0.02)	0.44 (0.05)	0.29 (0.06)
RFI_CT_lean	0.3 (0.05)	0.35 (0.07)	0.24 (0.05)	0.42 (0.08)	0.35 (0.09)
DFI	0.29 (0.03)	0.4 (0.04)	0.39 (0.02)	0.36 (0.05)	0.3 (0.06)
FPV	0.24 (0.03)	0.28 (0.03)	0.28 (0.02)	0.25 (0.04)	0.28 (0.06)
FR	0.56 (0.04)	0.59 (0.04)	0.57 (0.02)	0.52 (0.05)	0.46 (0.07)
NVD	0.21 (0.02)	0.27 (0.03)	0.31 (0.02)	0.31 (0.04)	0.19 (0.04)
TPV	0.32 (0.03)	0.4 (0.03)	0.37 (0.02)	0.4 (0.04)	0.24 (0.05)
TPD	0.49 (0.04)	0.53 (0.04)	0.54 (0.02)	0.47 (0.05)	0.44 (0.07)
DFI_night	0.27 (0.04)	0.38 (0.04)	0.43 (0.03)	0.37 (0.05)	0.21 (0.06)
TPD_night	0.26 (0.04)	0.38 (0.04)	0.43 (0.03)	0.36 (0.05)	0.24 (0.06)
NVD_night	0.27 (0.04)	0.38 (0.04)	0.45 (0.03)	0.34 (0.05)	0.26 (0.07)
FI23	0.18 (0.02)	0.22 (0.02)	0.27 (0.02)	0.26 (0.04)	0.15 (0.04)
FI235	0.31 (0.03)	0.31 (0.03)	0.32 (0.02)	0.34 (0.04)	0.39 (0.06)
BFT	0.59 (0.03)	0.64 (0.04)	0.63 (0.02)	0.63 (0.05)	0.39 (0.07)
LMD	0.38 (0.04)	0.36 (0.04)	0.39 (0.02)	0.21 (0.04)	0.46 (0.10)
IMF	0.29 (0.05)	0.23 (0.07)	0.22 (0.06)	0.71 (0.10)	0.25 (0.06)
LP	0.63 (0.05)	0.59 (0.07)	0.68 (0.07)	0.54 (0.07)	0.71 (0.10)

Bivariate animal models were conducted to estimate genetic correlations between traits across all breeds (Additional file 2, Figure S3). Most traits were positively correlated with RFI, except for FPV, FI235, and night FB traits (NVD_night, DFI_night, and TPD_night). Additionally, FPV and FI235 had positive genetic correlations with BFT, ADG, IMF, and LMD, but a negative genetic correlation with LP. Night FB traits had negative genetic correlations with production traits.

### Genome-wide association analysis and candidate gene identification

**Fig. 2:**
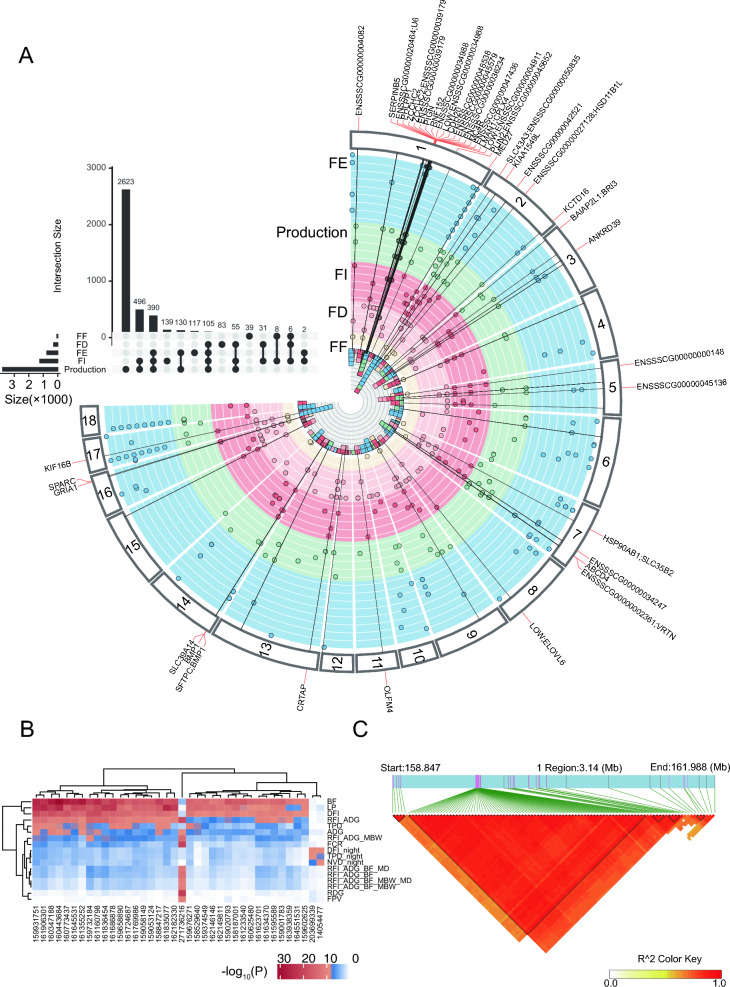
Genome-wide association results identifying significant pleiotropic loci across feed efficiency, feeding behavior, and production traits. **A** Fujiplot[84] showing significant SNPs, with ring colors indicating trait categories. Black lines highlight pleiotropic SNPs shared across traits. The UpSet plot summarizes the intersections of significant SNPs among trait categories. Left bar chart: the number of significant SNPs (y-axis) for each of the five trait datasets. Dot matrix: Dots indicate dataset membership in each intersection; connected dots represent shared membership across datasets. Horizontal bars (main plot): Length indicates the number of significant SNPs in each specific intersection combination. FE represents feed efficiency-related traits, including FCR, RFI_ADG, RFI_ADG_MBW, RFI_ADG_BF, RFI_ADG_BF_MBW, RFI_ADG_BF_MD, RFI_ADG_BF_MBW_MD, RFI_CT_lean, and RDG. Production represents growth and carcass traits, including ADG, BFT, LMD, IMF, and LP. FI represents feed-intake traits (DFI, DFI_night, FI235, FI23, FPV); FD represents feeding-duration traits (TPD_night, TPD, TPV, FR); and FF represents feeding-frequency traits (NVD, NVD_night). **B** Heatmap of -log10(P) values for all significant variants detected on SSC1. The x-axis shows SNPs that were significant for at least one trait, and the y-axis shows the corresponding traits. **C** LD block in the region SSC1:158,187,001–160,455,153

A total of 5,738 pigs were used as the discovery populations in the GWAS. Manhattan and QQ plots for each trait are in Fig. S4 and S5, respectively. A total of 1,749 associated markers and 358 independent markers were identified across traits (Fig. 2a and Additional file 1, Table S3, *P* < 4e−09). Interestingly, the 158,187,001–164,551,531 bp region on SSC1 was associated with multiple traits (Fig. 2a), including BFT, LP, ADG, DFI, RFI_ADG, and RFI_ADG_MBW (Fig. 2B and Additional file 1, Table S3). A narrow region from 159,658,890–160,953,032 bp was found to be in high LD (Fig. 2c). Importantly, SSC1:160,773,437 is the missense variant of *MC4R* that has been previously identified to be associated with BFT [50], IMF [50], ADFI [4], FCR [51], ADG [51], and ham weight [51]. Additionally, SSC1:271,736,216 and SSC1:271,736,217 were identified as pleiotropic for multiple traits, including the ADG, FPV, and all FE traits (except RFI_CT_lean) (Fig. 2A-B and Additional file 1, Table S3). These SNPs were intron variants of Mediator Complex Subunit 27 (*MED27*) gene, a key component of the eukaryotic transcription machinery that links transcription factors to the RNA polymerase II complex [52]. The PigBiobank [48] has reported an association of *MED27* with meat-to-fat ratio. Furthermore, a total of 19 missense variants across 7 genes were identified to be associated with traits (Table S3). For instance, SSC16:71,377,326, a missense variant of secreted protein acidic and cysteine rich (*SPARC*), exhibited associations with DFI_night (*P* = 9e−11), NVD_night (*P* = 9.37e−11), and TPD_night (*P* = 2.25e−10). SSC12:52,579,083, a missense variant of *ACADVL*, was associated with TPD_night (*P* = 2.77e−09). *ACADVL* encodes a protein involved in mitochondrial fatty acid beta-oxidation, which is crucial for energy release and body metabolism [53]. In total, 292 candidate genes were identified, including 139 for production traits, 65 for FE traits, and 114 for FB traits (49 for FD, 23 for FF, 83 for FI) (Table S3). Further annotation using KOBAS [47] (Additional file 2, Figure S6 and Additional file 1, Table S4) revealed that two genes, *RYR2* and *GRIA1*, were involved in the Circadian entrainment pathway. Notably, intron variants of *RYR2* at SSC14:54,227,097 (*P* = 2.52e−09) and SSC14:54,227,101 (*P* = 3.49e−09) were identified to be associated with DFI. An intron variant of *GRIA1* at SSC16:69,492,387, was identified to be associated with NVD_night (*P* = 1.07e−11), DFI_night (*P* = 1.40e−12), and TPD_night (*P* = 5.40e−12) (Fig. 2a and Additional file 1, Table S3).

### SNP selection based on GWAS summary data significantly improved GP accuracy

**Fig. 3:**
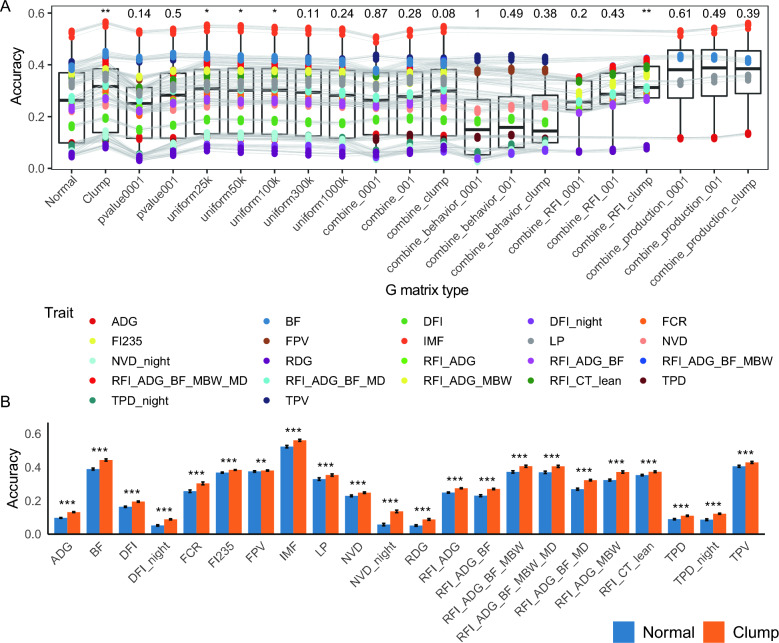
Genomic prediction (GP) accuracy in the validation group. **A** Boxplots comparing GEBV prediction accuracy across SNP datasets. ‘Normal’ denotes the standard GBLUP using the full set of genome-wide SNPs. The remaining panels on the x-axis represent SNP subsets selected based on GWAS significance and linkage disequilibrium criteria, with detailed definitions provided in Table 2. Asterisks above the bars indicate the statistical significance of differences in GP accuracy between each SNP preselection strategy and the ‘Normal’ (full SNP) model: *P < 0.05, **P < 0.01, ***P < 0.001. **B** GP accuracy by trait using Clump SNP sets. Asterisks above the bars indicate the statistical significance of differences in GS accuracy between ‘Clump’ and the ‘Normal’ (full SNP) model

One important application of GWAS summary data is enhancing GP accuracy in farm animals, which also validates the reliability of the GWAS results. In this study, we compared multiple GP scenarios based on SNP subsets derived from GWAS summary statistics, including SNPs selected by significance thresholds (*P* < 0.01 or *P* < 0.001), independent SNPs based on LD pruning (denoted as Clump), uniformly spaced SNPs across the genome (Uniform25k–Uniform1000k), and combined subsets of SNPs across all traits or specific trait categories, including feed behavior, feed efficiency, and production traits (Table 2). The ‘Clump’ method achieved the highest overall accuracy, with improvements of 13.7, 14.5, and 35%, for FE, ADG, and FB traits, respectively, and an average improvement of 13.4% (*P* = 0.007, two-tailed t-test) (Fig. 3b and Additional file 1, Table S5). This was followed by uniform25k (10.9% improvement, *P* = 0.02), uniform50k (10% improvement, *P* = 0.04), and uniform100k (9.5% improvement, *P* = 0.05), indicating that GP accuracy decreased as SNP density increased (Fig. 3a and Additional file 1, Table S5). This decline is likely due to the inclusion of more non-significant markers, supporting the reliability of the GWAS summary data. Considering the high genetic correlations among traits, we further tested whether combining SNP signals within the same trait category or across different trait categories could enhance GP accuracy. We created combined subsets for each trait category. However, only the combined SNPs from feed efficiency traits (combine_rfi_clump) resulted in a significant overall improvement in GP accuracy (*P* < 0.01), while combining SNPs from other major trait categories did not produce significant gains (Fig. 3a).

### Post-GWAS analysis using *cis*-eQTL

To further elucidate the biological mechanisms underlying our GWAS findings, we performed several in silico analyses to functionally annotate and prioritize the most likely causal genes for significant SNPs. We conducted TWAS for 34 tissues based on the GWAS results and integrated these findings with COLOC [40] and SMR [41] analyses to prioritize target genes.

**Fig. 4:**
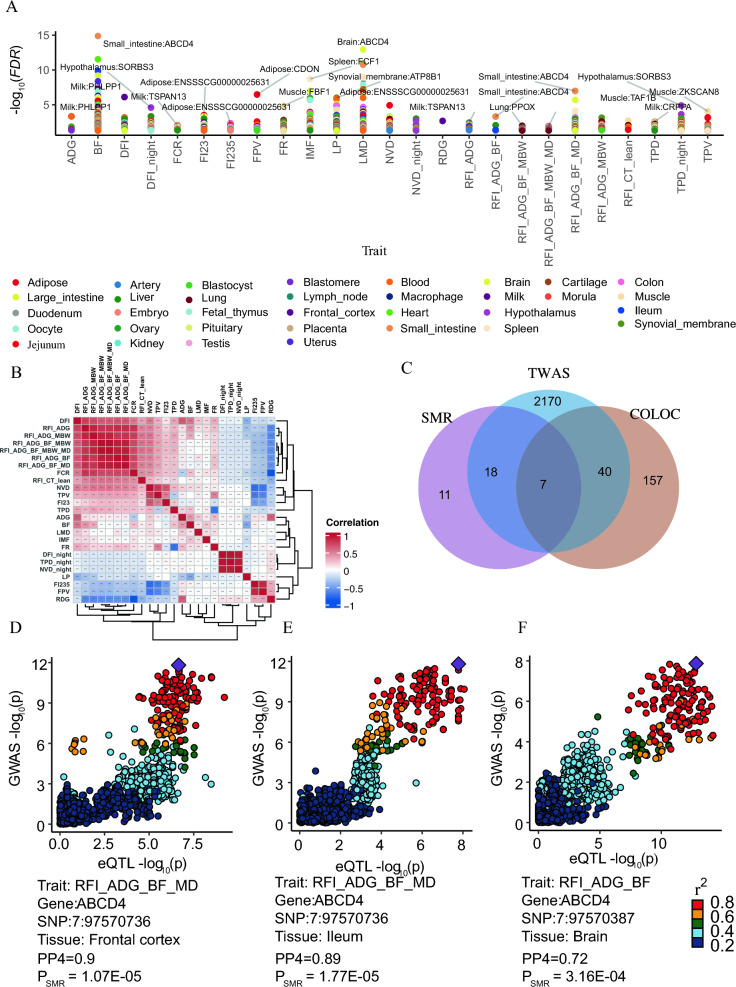
Post-GWAS results. **A** Scatterplot of tissue-gene pairs associated with traits by TWAS (FDR ≤ 0.05), colored by tissue. **B**The heatmap displays pairwise correlations among traits calculated from genome-wide TWAS Z-score profiles. For each trait pair, Pearson correlation coefficients were computed using the Z-scores of all tested genes, generating a correlation matrix that reflects similarity in gene–trait association patterns across traits. Warmer colors indicate stronger positive correlations, whereas cooler colors represent negative or weaker correlations. **C** Venn diagram of candidate tissue-genes from three methods (TWAS/COLOC/SMR). **D–F** Scatterplots showing correlations between *cis*-eQTL of *ABCD4* (in different tissues) and GWAS results for RFIs. LD (r^2^) values between eVariant and surrounding SNPs are color-coded

**Fig. 5:**
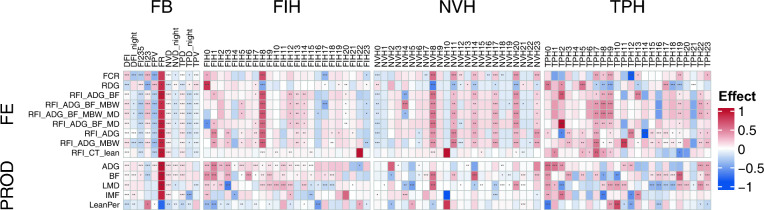
One-sample Mendelian randomization (MR) results between feeding behavior traits and growth/feed efficiency traits. The heatmap shows the MR-estimated effect sizes, with colors representing the direction and magnitude of the effects. Asterisks indicate statistical significance: *P ≤ 0.05, **P ≤ 0.01, ***P ≤ 0.001. Results without asterisks are non-significant

A total of 2235 trait-tissue-gene (*FDR* < 0.05) pairs were identified in TWAS analyses (Fig. 4a and Additional file 1, Table S6). Additionally, 65 putative causal trait-tissue-gene associations met at least two criteria, the criteria have been described in methods, with seven supported by all three criteria (TWAS, COLOC, SMR) (Fig. 4c and Additional file 1, Table S7). *ABCD4* was identified to be associated with RFIs by TWAS (trait: RFI_ADG_BF_MD, tissue: small intestine, *Z* = −7.06, *P* = 1.7e−12, *FDR* = 1.01e−07). It was further identified to be significantly associated with FE by the COLOC and SMR analyses (Fig. 5d-f and Table S7) in the brain (*FDR*_*TWAS*_ = 0.008, *PP4*_*COLOC*_ = 0.72, *FDR*_*SMR*_ = 0.49), frontal cortex (*FDR*_*TWAS*_ = 0.0007, *PP4*_*COLOC*_ = 0.89, *FDR*_*SMR*_ = 0.054), ileum (*FDR*_*TWAS*_ = 0.005, *PP4*_*COLOC*_ = 0.89, *FDR*_*SMR*_ = 0.056), blood (*FDR*_*TWAS*_ = 0.002, *PP4*_*COLOC*_ = 0.62, *FDR*_*SMR*_ = 0.008), and adipose (*FDR*_*TWAS*_ = 0.036, *PP4*_*COLOC*_ = 0.91, *FDR*_*SMR*_ = 0.63). These results indicates that *ABCD4* was a causal gene for FE traits. *ABCD4* was also associated with both BFT and LMD (Table S7). FHF Complex Subunit HOOK Interacting Protein 2B (*FHIP2B)* was identified as a candidate gene for DFI_night (*FDR*_*TWAS*_ = 0.006, *PP4*_*COLOC*_ = 0.86, *FDR*_*SMR*_ = 0.02), TPD_night (*FDR*_*TWAS*_ = 0.006, *PP4*_*COLOC*_ = 0.79, *FDR*_*SMR*_ = 0.017) and NVD_night (*FDR*_*TWAS*_ = 0.04, *PP4*_*COLOC*_ = 0.89, *FDR*_*SMR*_ = 0.13) in blood.

To further explore the relationships between traits at the gene expression level, we calculated Pearson correlation coefficients between the traits TWAS z-scores. This allowed us to assess how genetically regulated expression of gene is shared across traits. The results revealed that FPV, FI235, and night FB were negatively correlated with FE traits, aligning with the findings from the genetic correlation analysis (Figs. 4b and 1e).

### Mendelian randomization revealed causal effects

To investigate the relationship of FB with other traits, we conducted one-sample MR analyses (Fig. 5 and Table S8). FB traits consistently showed the same direction of effects across various RFI measurements and FCR (Fig. 5 and Additional file 1, Table S8). Notably, FPV (effects: −0.84 ~ −0.70, *P* < 0.001) and FI235 (effects: −0.52 ~ −0.24, *P* < 0.001) had negative impacts on RFIs (Fig. 5 and Table S8), while FI23 exhibited positive effects on RFIs, consistent with the results from genetic correlations and TWAS results. Increasing FPV (effects: 0.15, *P* = 9.85E-05) and FI235 (effects: 0.14, *P* = 2.04E−10) was also estimated to increase ADG (Fig. 5). Interestingly, DFI_night, TPD_night and NVD_night demonstrated a negative causal relationship with RFIs, while DFI, TPD, and NVD exhibited positive effects (Fig. 5 and Table S8). To further investigate the effects of FB traits at different times of the day, we conducted MR analysis of hourly FB traits with FE and production traits. Significant negative causal relationships with RFIs were observed for FIH16, FIH17, FIH23, NVH21, NVH22, NVH0, TPH19, and TPH2, while FB during other times displayed a positive relationship with RFIs (Fig. 5 and Table S8). This aligned with the negative causal relationship of night FB traits with FE traits. Additionally, FIH8, NVH8, TPH7-9 affected the RFIs significantly (Fig. 5 and Table S8), corresponding to peak FB periods in the morning (Fig. 1b-d), whereas FB traits during the afternoon peak period showed weaker associations with RFI. Furthermore, MR analysis between FB and production traits (ADG, BFT, LMD, IMF and LP) revealed that DFI_night, NVD_night, and TPD_night had higher effects on LP than DFI, NVD, and TPD (Fig. 5 and Additional file 2, Figure S7). This is consistent with findings in humans, where night eating behaviors have been associated with increased obesity [54–56].

### Genetic links between pig feeding-related traits and human traits

**Fig. 6:**
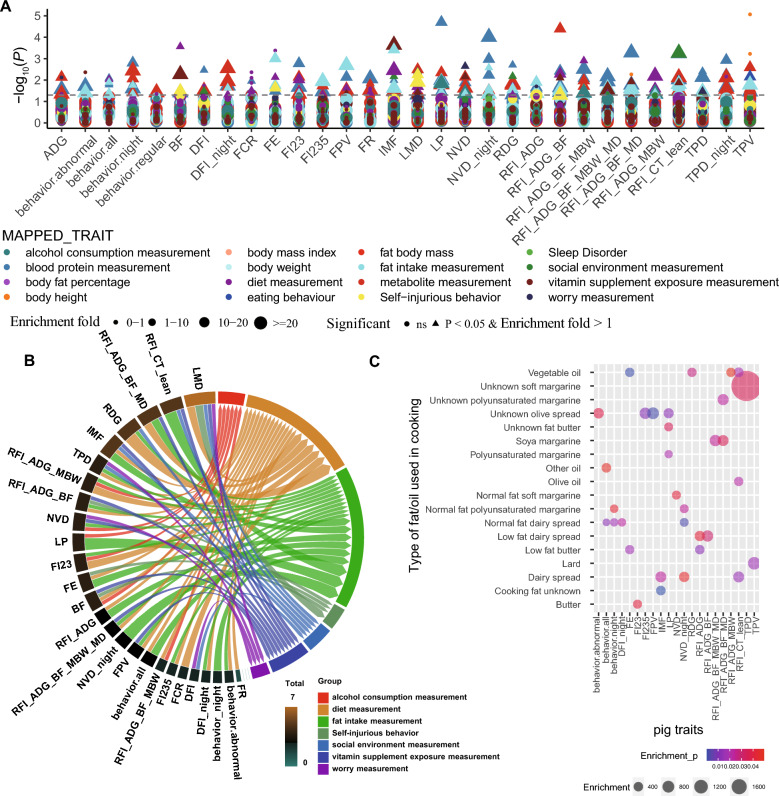
Comparative analysis of human and pig traits. **A** Heritability enrichment of pig feed-related GWAS in 205 human GWAS. The x-axis shows pig traits, y-axis shows heritability enrichment significance. Dot colors indicate human trait categories; size indicates enrichment fold. Triangles represent significant traits; circles represent non-significant traits. **B** Chord diagram illustrating associations between pig and human trait categories. The left semicircle represents two pig traits. The right semicircle corresponds to broad categories of human diseases/traits from GWAS. Ribbons connecting sectors indicate the number of trait pairs showing significant heritability enrichment, with ribbon width proportional to the count of significant associations. The length of each sector reflects the total number of significantly enriched human GWAS traits linked to the respective pig trait category. **C** Heritability enrichment between FB traits and human fat intake. The horizontal axis represents pig traits, while the vertical axis corresponds to human fat intake-related. The size of each intersection point indicates the enrichment fold of heritability, and the color gradient reflects the *P* value

To investigate the association of pig feeding-related traits with human health traits, we conducted heritability enrichment analyses. We identified 247 pig-human trait pairs showing significant enrichment of SNP-based heritability for human traits within genomic regions associated with pig feeding-related traits (*P* < 0.05 and enrichment fold > 1), representing 146 distinct human traits across seven categories (Fig. 6a-b and Additional file 1, Table S9). The most enriched categories of human traits were fat intake and diet (Fig. 6b). Further analysis using Fisher’s test revealed significant enrichment of pig traits such as NVD_night, behavior_night, RFI_CT_lean, and LP in the fat intake category (*P* < 0.05) (Additional file 1, Table S10). Interestingly, variants associated with DFI_night and NVD_night were enriched in traits related to margarine (normal fat soft margarine, normal fat polyunsaturated margarine) and fat (normal fat dairy spread, dairy spread) used in cooking, but not with the use of olive or vegetable oil (Fig. 6c). Fat intake is crucial in human health, as higher intake of butter and margarine has been associated with increased mortality, while canola and olive oil intake has been linked to reduced mortality [57]. Additionally, margarine consumption was reported to increase triacylglycerols (TGs), glycosylated hemoglobin (HbA1c), and isoprostanes (IsoPs) values in mice [58], aligning with the finding that night feeding behavior reduces LP. These results indicate that genes associated with pig feeding behavior may be related to human health, encouraging further investigation into the behavioral connections between humans and pigs in future studies.

## Discussion

Although feed constitutes the largest proportion of pig production costs, research on FE remains limited, primarily due to the challenges in accurate measurement. To our knowledge, this is the largest GWAS study to date that employs multi-ancestry breeds to investigate 28 pig feeding-related traits (n = 5738). We identified 358 independent variants associated with these traits and, by leveraging the GWAS summary data, we significantly improved GP accuracy in the validation population (n = 1200). We acknowledge that combining multiple breeds introduces heterogeneity in allele frequencies, LD patterns, and genetic backgrounds, which may affect heritability estimates and model performance. Nevertheless, by integrating multiple breeds, we were able to identify loci that are consistently associated with feeding behavior and efficiency across populations, which in turn contributed to measurable improvements in GP accuracy in the validation cohort. Furthermore, while the influence of FB on FE has been described previously, most previous studies are confined to correlation analyses. This study is the first to utilize MR to elucidate the relationships between FB, FE, and production traits. Our findings underscore the critical role of FB in enhancing FE and production traits, with notable effects observed in FPV, FI235, and night FB. Additionally, our results revealed genetic associations between pig FB traits and human dietary habits, including fat intake and diet, indicating the potential application of the pig as a model in human health. These results will encourage researchers to explore further connections between pig and human traits using the extensive data recorded in pig breeding practices.

### Integrative post-GWAS analyses identify *ABCD4* as a candidate gene

The availability of limited genomic data makes it difficult to identify causal genes of feed-related traits. Our analysis integrated *cis*-eQTL from 34 PigGTEx tissues [10]. Through the application of three methods (TWAS, COLOC, SMR), we identified the *ABCD4* gene as a significant candidate gene for RFI, as its expression levels in the brain and frontal cortex were strongly associated with RFI. The ABCD4 protein is known for its role in transporting vitamin B12 (cobalamin) from the lysosomal lumen to the cytosol, a process coupled with ATP binding and hydrolysis [59, 60]. Vitamin B12 is essential for neuronal health, such as the maintenance of the myelin sheath [61]. Pathogenic variants in *ABCD4* can lead to inborn errors in cobalamin metabolism, resulting in conditions such as methylmalonic aciduria and homocystinuria [62, 63]. These findings underscore the critical role of *ABCD4* in neurological and metabolic health, consistent with the important role of *ABCD4* highlighted by our post-GWAS integrative analyses.

### Causal relationships between feeding behavior and feed efficiency

The relationship between FB and FE traits has been extensively studied. Our study further explored the causal relationship across a multi-breed population using MR, using seven RFI adjustment methods, which will help researchers compare their results with those of this study. Notably, we observed that incorporating MBW as a covariate slightly reduced the heritability of RFI. This is likely because adjusting for MBW removes a portion of the genetic variance shared with feed intake. Conceptually, this aligns with the original definition of RFI proposed by Koch et al. [64], in which RFI is intended to reflect feed intake independent of metabolic body weight. For instance, fewer NVD [13] and lower DFI [65] have been reported to be phenotypically associated with improved feed efficiency, which aligned with our results. Interestingly, our study revealed that higher FPV and FI235 are genetically associated with improved FE, highlighting their potential value in breeding. This conclusion was supported by three sets of results: estimates of genetic correlations, Pearson correlations of TWAS z-score, and MR analysis. These findings suggest that optimizing FPV and FI235 could help improve FE without compromising growth rates. Furthermore, we identified loci significantly associated with both FPV and RFIs. For instance, variants SSC1:271,736,216 and SSC1:271,736,217, located within introns of the *MED27* gene, were associated with six RFIs, FCR, FPV, and TPV. A variant at SSC3:65,795,646 was identified to be associated with FI235, FPV, FCR, and TPV. Variants at SSC3:65,795,640 and SSC3:65,795,635 were associated with FPV and FCR. These SNPs (SSC3:65,795,635–65,795,646) reside in an intergenic region proximate to ENSSSCG00000042171 (122,929 bp away) and the leucine rich repeat transmembrane neuronal 4 gene (*LRRTM4*, SSC3:66,128,689–66,884,740). The pig RNA ATLAS [49] (www.rnaatlas.org) indicates that the ENSSSCG00000042171 gene is enriched in bone marrow and lymphoid tissue and is clustered in the immune cells, suggesting its potential regulatory role in RFI and FB traits through the immune system. Additionally, *LRRTM4* is enriched in the brain and retina, as shown by both pig RNA ATALS and the human protein atlas [66] (proteinatlas.org). *LRRTM4* has been identified as a candidate gene associated with herding, predation, and temperament behaviors in dogs [67], as well as with various human traits such as aggressive behavior [68], attempted suicide [69], autism spectrum disorder [70], intellectual disability [71], and bipolar disorder [72]. These findings indicate that *LRRTM4* has pleiotropic effects in the nervous system and may influence feeding-related behaviors through neural pathways.

### Night feeding behavior and its relevance to circadian biology and human health

We also investigated the impact of FB traits during the night. Our results proposed that night eating decreases the LP, consistent with studies linking night eating to obesity in humans [54–56]. In human studies, one diagnostic criterion for identifying individuals with night eating syndrome (NES) is the consumption of at least 25% of food after the evening meal or a minimum of two episodes of night eating per week [73]. In our study, pigs of the D2 breed exhibited an average of 29% of feed intake and 30% of feeding counts and time feeding during the night, whereas other breeds averaged about 20% (Table S1). We also identified 38 candidate genes associated with night FB traits. For instance, *ATP1A1* expression exhibited circadian variation, with maximal expression at circadian time 12 in rats fed ad libitum and decreased expression during restricted feeding phases [74]. In human, *CDKAL1* has been linked to external eating and metabolic syndrome [17], *ACADVL* to dietary fat intakes [75] and *PLIN2* to cardiac lipid accumulation [76]. *HSP90AB1* was responsible for maintaining proper cellular levels of the BMAL1 protein, influencing the mammalian circadian clock [77]. The *ELOVL6* gene codes a lipogenic enzyme that is regulated by fasting and refeeding [78, 79]. These findings suggest that night FB in pigs could serve as a valuable model for exploring eating behaviors and circadian rhythms in humans.

The rising obesity crisis and increasing cases of eating disorders, such as binge eating and anorexia, highlight the urgent need to understand feeding behavior mechanisms in humans [80, 81]. However, studying human eating behavior is challenging due to the reliance on subjective questionnaires, which can introduce biases. In contrast, pig FB is objectively and accurately measured through automated systems, providing comprehensive data. Pigs share similarities with humans in gastrointestinal function and composition, as well as in appetite regulation, with comparable blood hormone levels, including cholecystokinin, glucagon-like peptide-1, peptide YY, and ghrelin [1]. In nutritional neuroscience, the anatomical and developmental similarities between pig and human brains offer valuable insights into the relationship between nutrition, brain development, and metabolism [82]. Xu et al. [11] compared 136 human complex phenotypes with 232 pig complex traits but faced significant limitations due to small sample sizes and the limited number of feeding-related traits analyzed. Do et al. [83] have reported that genomic regions associated with pig FB may be associated with eating behavior and obesity in human beings. In this study, we conducted trait mapping using heritability enrichment analysis across 16 categories of human traits. Our results revealed that pig FB traits were associated fat intake in humans. These findings highlight the important role of FB in human health.

### Limitations and future perspectives

Our study has several limitations. We prioritized candidate genes using publicly available PigGTEx cis-eQTL data, which were not generated in this study, and these genes were not experimentally validated at the molecular or animal level. While this approach provides valuable insights, integrating additional transcriptomic or single-cell data and performing functional validation could further strengthen the interpretation of our results. Additionally, although we have established preliminary correspondences between pig feeding behavior and human traits, we lack detailed data to further explain the underlying molecular mechanisms. We map pig genomic regions to humans using 1 Mb windows, an approach that may be affected by future updates to reference genome assemblies, which could alter genomic coordinates, orthologous mappings, and the annotation of corresponding regions. Therefore, these results should be considered as suggestive of a potential link between human and pig feeding behaviors, and further investigation will be required. Future research should focus on collecting multi-omics data from various tissues of both pigs and humans and conducting homologous analysis at the molecular level to elucidate the intrinsic mechanisms.

## Conclusions

This study provides a comprehensive dissection of the genetic architecture underlying pig feeding behavior, feed efficiency, and production traits. By performing multi-ancestry GWAS on feeding-related traits and integrating cis-eQTL data from pigGTEx, we identified 358 independently associated variants and highlighted a pleiotropic QTL on SSC1 for FE, FB and production traits. Genomic prediction analyses demonstrated that leveraging these GWAS results can significantly enhance prediction accuracy, supporting practical breeding applications. Mendelian randomization further revealed causal relationships between specific feeding behaviors and feed efficiency or growth traits, while heritability enrichment analyses suggested potential cross-species links between pig feeding traits and human metabolic and dietary traits.

Collectively, these findings advance our understanding of the genetic regulation of feed efficiency, provide valuable resources for genomic prediction in pigs, and offer novel insights into conserved mechanisms that may influence feeding and metabolic phenotypes across species. Future work integrating multi-omics data and functional validation will further refine these candidate genes and causal pathways, ultimately informing both animal breeding strategies and comparative studies of feeding behavior.

## Supplementary Information

Additional file1 (DOCX 2171 KB)

Additional file2 (XLSX 1687 KB)

## Data Availability

The GWAS summary statistics of this study are openly available at https://alphaindex.zju.edu.cn/ALPHADB/download.html
