# Supplementary material for: Integrating GWAS and eQTL analysis to decipher genetic mechanisms of feed efficiency and feeding behaviors in pigs
Source: Genet Sel Evol. 2026 Apr 26;58:31. doi: 10.1186/s12711-026-01048-7 (PMC13262426; doi:10.1186/s12711-026-01048-7)
Supplement: Supplementary file 1 — Additional file1 (DOCX 2171 KB) [file 12711_2026_1048_MOESM1_ESM.docx]

**Supplemental Figures**


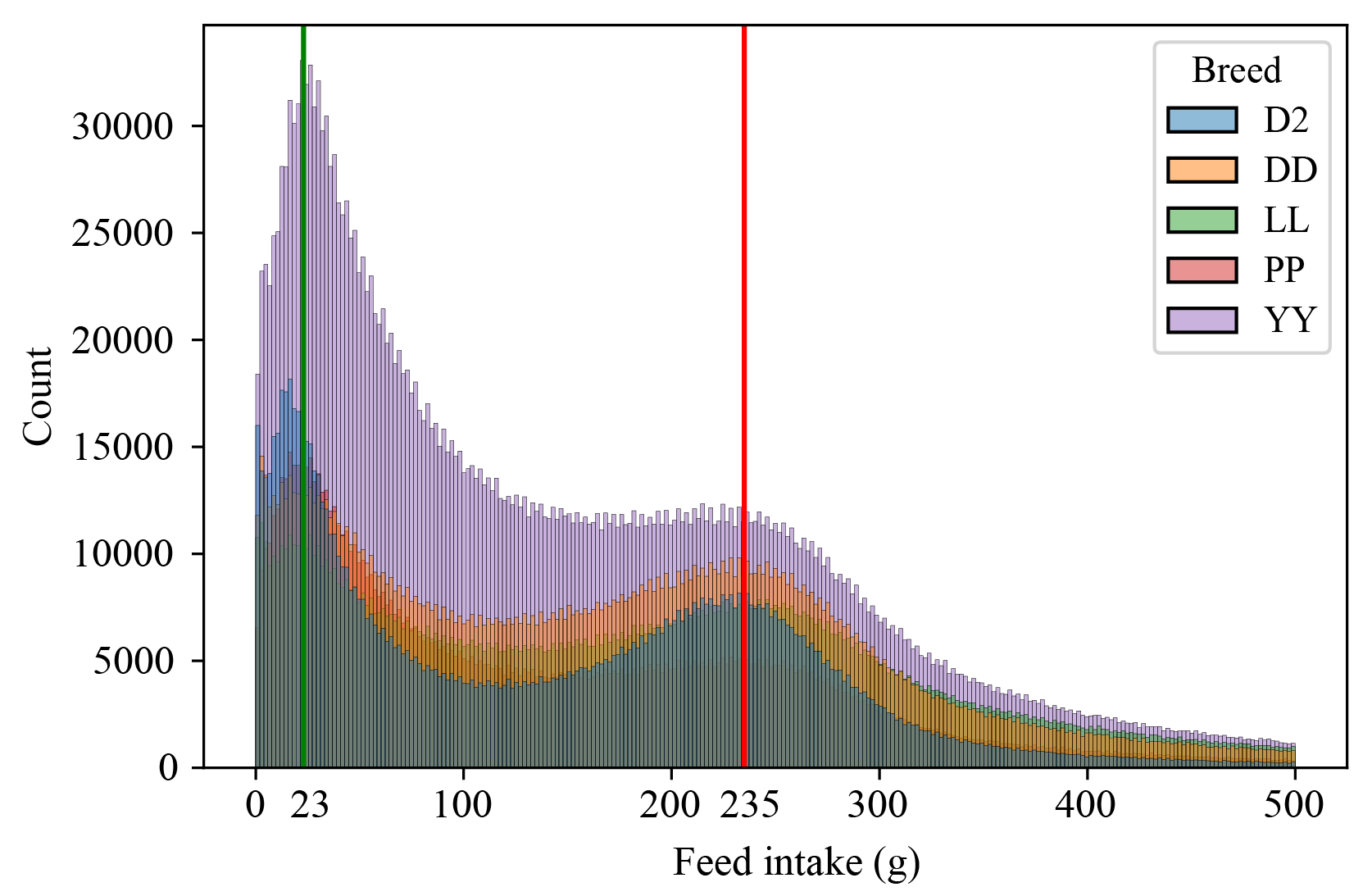


**Supplementary Figure S1. The distribution of feed intake.** The x-axis represents the feed intake per visit for each individual, and the y-axis shows the corresponding frequency. Different colors indicate various breeds. The green vertical line marks the first peak, while the red vertical line marks the second peak. D2: Duroc2 (another Duroc line characterized by high intramuscular fat content); DD: Duroc; LL: Landrace; YY: Yorkshire; PP: Pietrain.

**
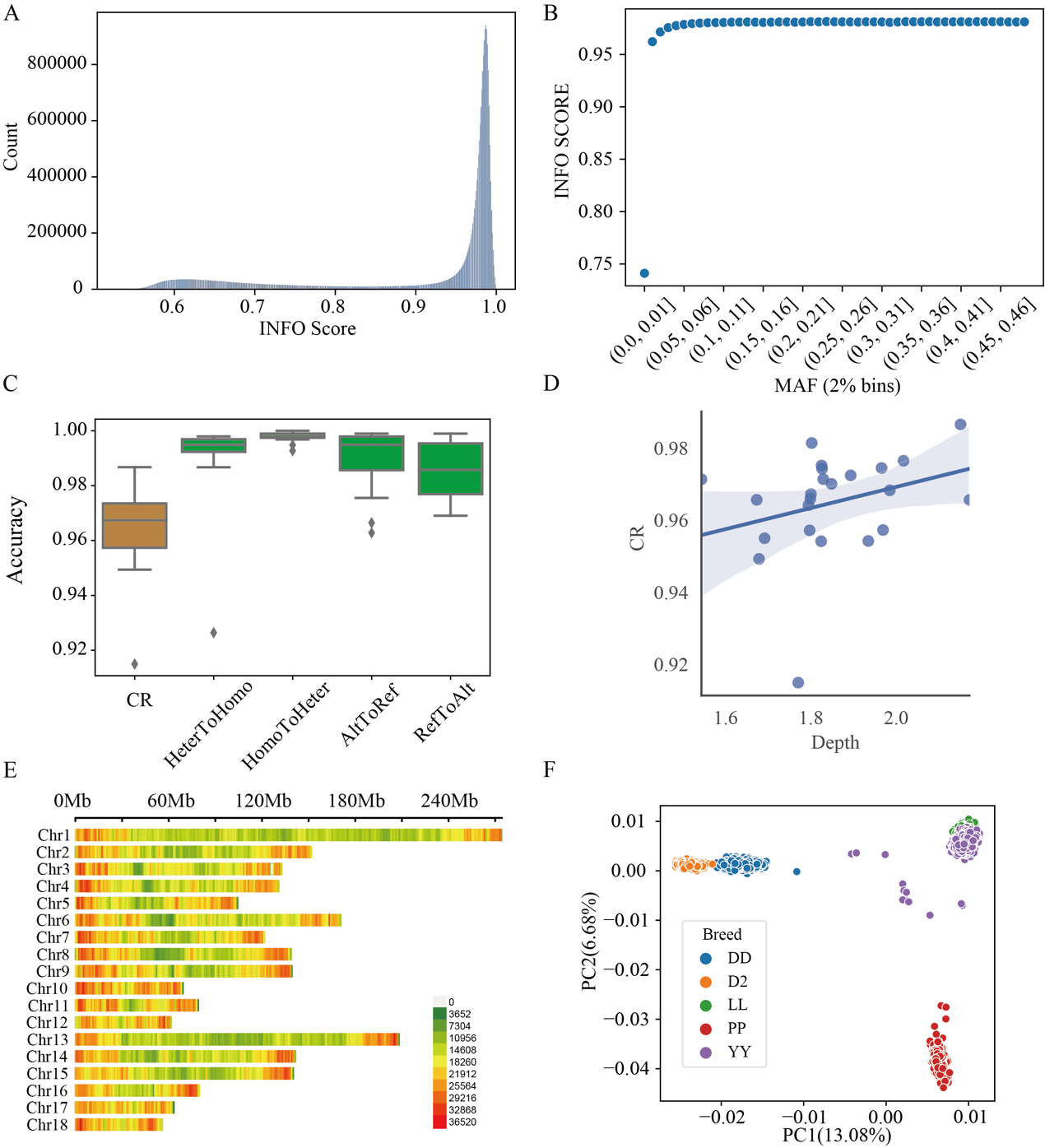
**

**Supplementary Figure S2. The genome imputation accuracy. (A)** Bar plot showing the frequency of the INFO score. **(B)** Scatter plot showing the correlation between INFO score and MAF, the x-axis is the MAF bins, and y-axis is the INFO score. **(C)** Box plot showing the imputation accuracy, estimated by imputed SNPs and SNPs identified by GGP 50K chip. The y-axis is the value, x-axis represents the estimated types. CR represents the overall consistency rating between the imputed SNPs and chip. HeterToHomo represents the accuracy heterozygote to homozygote, the value is calculated as the 1 – the rate of the heterozygote imputed as homozygote. HeterToHomo represents the accuracy homozygote to heterozygote, the value is calculated as the 1 – the rate of the homozygote imputed as heterozygote. AltToRef represents the accuracy of the alt allele. RefToAlt represents the accuracy of the ref allele. **(D)** Scatterplot plot showing the correlation between CR and depth. Each point represents each pig. **(E)** SNP density after imputation. **(F)** The population structure showed by the PCA analysis.


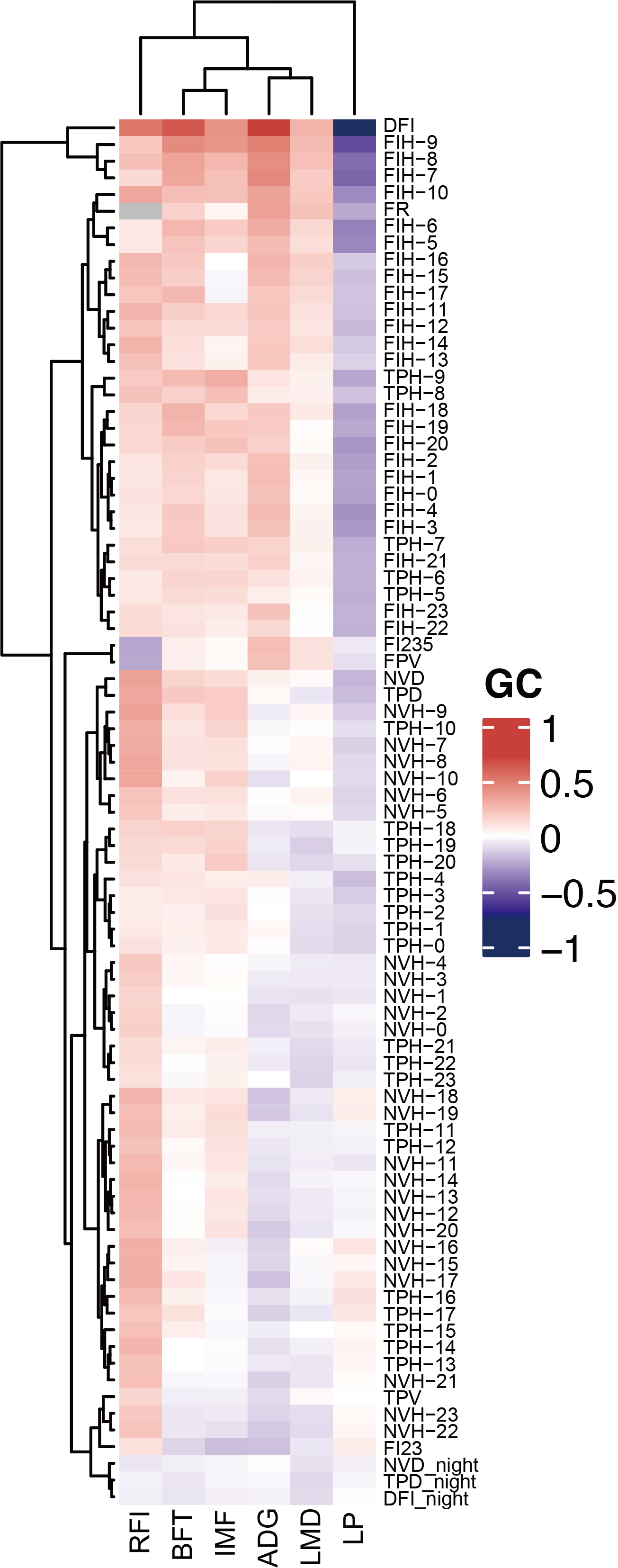


**Supplementary Figure S3. Genetic correlations between feeding behavior traits and feed efficiency (RFI_ADG_BF_MBW_MD), BFT, IMF, ADG, LMD, and LP.** FIH-[0–23] represents hourly feed intake from 0:00 to 23:00, TPH-[0–23] represents hourly feeding time, and NVH-[0–23] represents hourly feeding frequency.


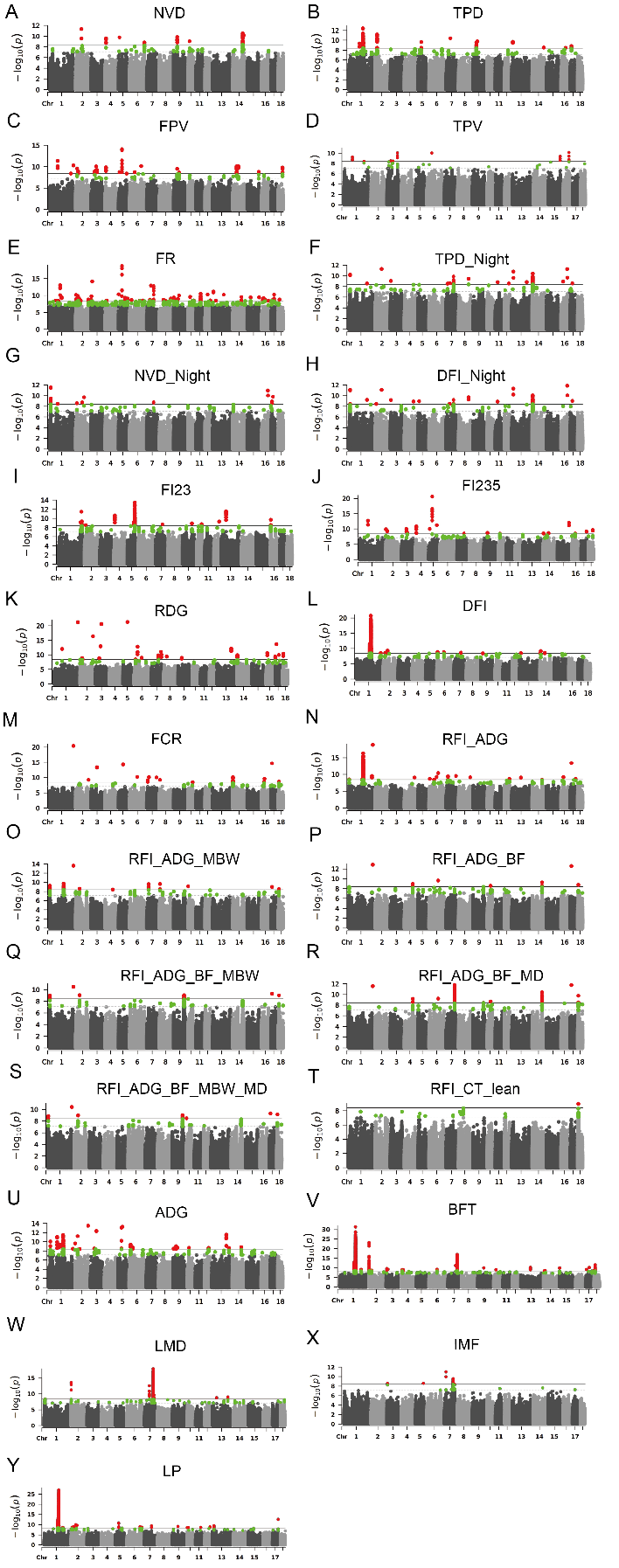


**Supplementary Figure S4. The Manhattan plots of all the traits GWAS.** The x-axis represents the chromosomes, and the y-axis shows the -log_10_(*P*) value for each point. Red dots denote points reaching the significance threshold, green dots indicate points that have reached the suggestive significance threshold.


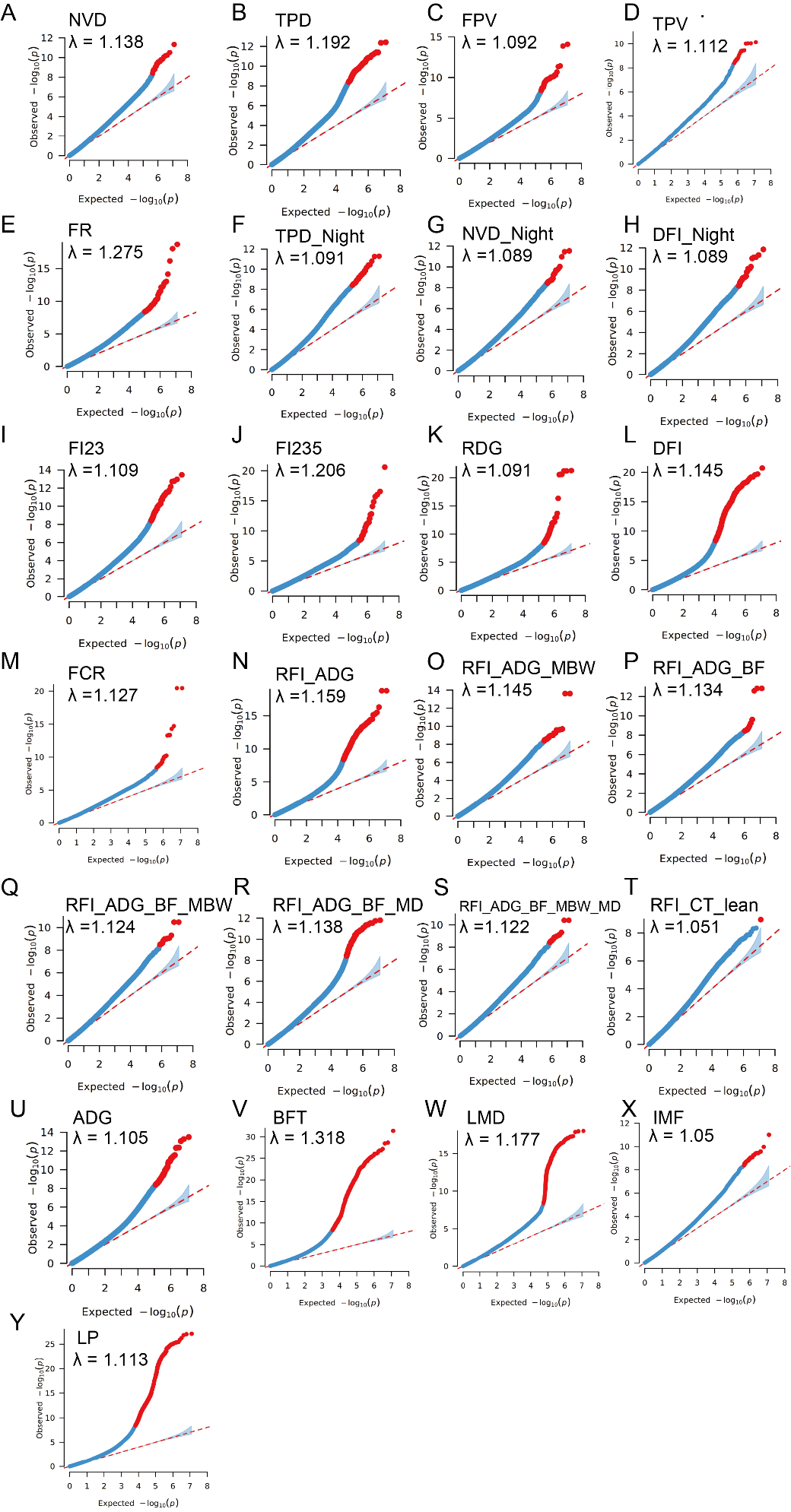


**Supplementary Figure S5.** The QQ plots of all the traits GWAS. Red dots indicate points where the p-value has reached the suggestive significance threshold.


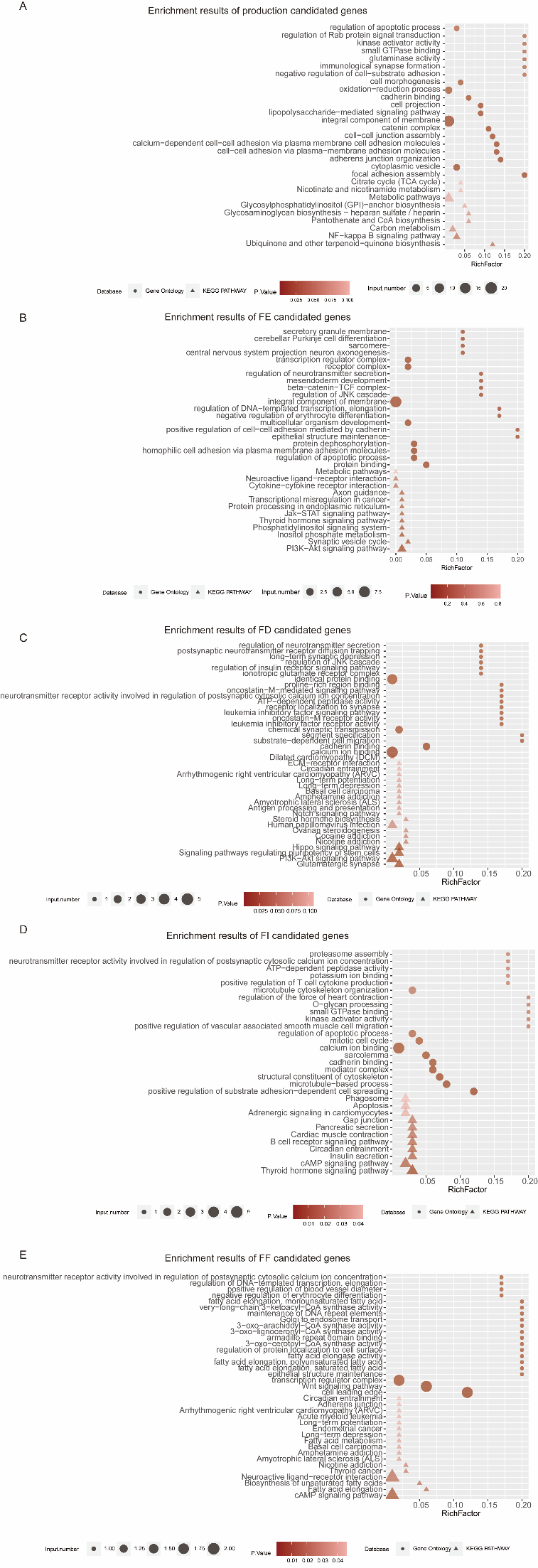


**Supplementary Figure S6.** Results of GO and KEGG enrichment analysis by KOBAS for the candidate genes of production traits(A), FE traits (B), FD traits (C), FI traits (D), FF traits(E)


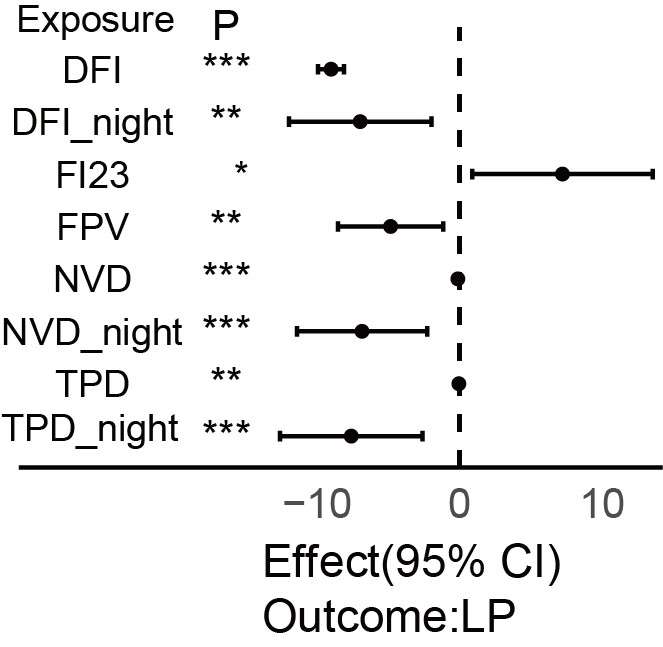


**Supplementary Figure S7.** Forest plot showing the effects of feeding behavior to Lean meat percentage.
